# The use of dupilumab in children and adolescents with severe atopic dermatitis: a systematic review and meta-analysis

**DOI:** 10.1590/1806-9282.2024D711

**Published:** 2025-03-17

**Authors:** Maria Fernanda Mollaco Navarro da Cruz, Luca Schiliró Tristão, Clara Lucato dos Santos, Sandra Lopes Mattos e Dinato, Wanderley Marques Bernardo

**Affiliations:** 1 Centro Universitário Lusíada, Faculty of Medical Sciences of Santos, Department of Evidence-Based Medicine – Santos (SP), Brazil.; 2 Brazilian Medical Association, Department of Evidence-Based Medicine – São Paulo (SP), Brazil.; 3 Centro Universitário Lusíada, Department of Dermatology – Santos (SP), Brazil.; 4 Universidade de São Paulo, Faculty of Medicine – São Paulo (SP), Brazil.

The Guidelines Project, an initiative of the Brazilian Medical Association, aims to combine information from the medical field to standardize how to conduct, and to assist in the reasoning and decision-making of doctors. The information provided by this project must be critically evaluated by the physician responsible for the conduct that will be adopted, depending on the conditions and the clinical condition of each patient.

## DESCRIPTION OF THE EVIDENCE COLLECTION METHOD

A systematic review of the literature was conducted using the Medline, Embase, ClinicalTrials, and Google Scholar databases. The following search strategy was used: (Dermatitis, Atopic OR Atopic Dermatitides OR Atopic Dermatitis OR Atopic Neurodermatitides OR Atopic Neurodermatitis OR Disseminated Neurodermatitides OR Disseminated Neurodermatitis OR Atopic Eczema) AND (dupilumab) AND Random*.

A total of 533 publications were retrieved (with limits for randomized clinical trials and children), from which 58 papers were initially selected, meeting the eligibility criteria, and 3 studies were included to support this evaluation. The main reasons for excluding the 55 studies were as follows: systematic review (N: 5); head to head with biological (N: 1); observational (N: 2); post hoc (N: 11); pooled analysis (N: 12); pharmacokinetics (N: 2); without dupilumab (N: 1); intermediate endpoints (N: 5); without placebo (N: 3); letter to the editor (N: 1); phase II clinical trial (N: 5); commentary (N: 1); and other (N: 6).

## QUALITY OR CERTAINTY OF EVIDENCE

The quality of evidence of this review was expressed as very low, low, moderate, and high. Items considered (using the GRADEpro software) were rated as very high, high, and low, considering the following criteria: risk of bias, inconsistency, precision, indirect evidence, and publication bias.

## GOALS

To assess the efficacy and safety of dupilumab in the treatment of atopic dermatitis in children aged 6 months to 18 years

## INITIAL CONSIDERATIONS

Atopic dermatitis (AD) is a chronic clinical condition that produces dry, itchy, and inflamed skin. It is common in young children but can occur at any age. In these patients, comorbidities such as food allergies, hay fever, and asthma are commonly observed.

Symptoms of AD can appear anywhere on the body and vary widely from person to person. These may include dry, cracked skin; itching (pruritus); rash; oozing and crusting; thickened skin; darkening of the skin around the eyes; and sore, tender skin due to scratching.

Evaluating the severity of AD is essential for choosing the right treatment and for continuous monitoring as the therapeutic response may vary over time. There are several assessment instruments applied clinically or in research, measuring various aspects involved in the disease^1^:

- Body surface area (BSA): Assessment of the percentage of body area involved, not incorporating the severity of the lesion.
- Eczema Area and Severity Index (EASI): Scoring system that rates the area and severity of eczema in four regions of the body, with a total range of 0–72 points.
- Patient-Oriented Eczema Measure (POEM): Seven-item questionnaire assessing specific symptoms during the past week, with each item scored from 0 (no days) to 4 (every day) based on the number of days affected.
- Dermatology Life Quality Index (DLQI): Ten-item questionnaire assessing health-related quality of life in the past week, with each item scored for impact from not at all (0) to a great deal (3).
- Patient Global Impression of Severity—Atopic Dermatitis (PGI-S-AD): This is a single item that asks the patient how he or she would rate his or her symptoms over the past 24 h. The five response categories range from "no symptoms" to "severe."
- Investigator global assessment (IGA): This is a scale that is used to assess the overall severity of lesions at a given time. It is scored from 0 (clear) to 4 (severe) based on four clinical features of AD lesions: Erythema, induration/papulation, lichenification, and exudation/crusting, and takes into account the extent of the disease.
- Peak Pruritus Numerical Rating Scale (NRS): This is a single item used to measure peak pruritus, or "worst" itch, over the past 24 h based on the following question: "On a scale of 0 to 10, with 0 being "no itch" and 10 being "the worst itch imaginable," how would you rate your itch at its worst in the past 24 h?"

Glucocorticoids are currently the only approved systemic treatment for AD in children under 6 years of age, but they are not recommended by guidelines due to safety concerns. Currently available systemic therapies are used off-label in this population, with no clinical trial data to guide optimal use. Additionally, these medications pose significant safety concerns that limit their use^2^.

Despite the chronic nature of AD, treatment in children is often limited to short-term TCSs, with topical calcineurin inhibitors as a second-line therapy. Guidelines discourage the systemic use of corticosteroids due to the risk of relapse after a short-term treatment, an unfavorable benefit–risk ratio, and multiple AEs associated with their use^3^. While other systemic agents like cyclosporine have been used off-label, the risk of serious AEs and the lack of strong evidence supporting long-term efficacy make them particularly unsuitable for the 6–11-year-old age group. As a result, systemic treatments are reserved as a last resort for the most difficult cases, highlighting the significant need for better options for children whose disease remains inadequately controlled with topical therapy.

One of the systemic treatment options currently under study for children with severe AD that is inadequately controlled by topical therapies is dupilumab. This fully human drug, derived from a monoclonal antibody, blocks the receptors for interleukin-4 and interleukin-13^4^. Clinical trials with Dupilumab have shown that these cytokines are key drivers of multiple type 2 inflammatory diseases. Its use is approved in the United States, the European Union, and other countries for adults and adolescents with moderate to severe AD and moderate to severe asthma, and in adults with chronic rhinosinusitis and nasal polyps.

## OBJECTIVE

The objective of this study was to assess the efficacy and safety of dupilumab in the treatment of AD in children aged 6 months to 11 years and in adolescents.

## METHODS

This systematic review and meta-analysis followed the PRISMA^5^ precepts and is registered with PROSPERO^6^ under registration CRD42024585551.

A systematic review of the literature was conducted using the Medline, Embase, ClinicalTrials, and Google Scholar databases. The following search strategy was used:

(Dermatitis, Atopic OR Atopic Dermatitides OR Atopic Dermatitis OR Atopic Neurodermatitides OR Atopic Neurodermatitis OR Disseminated Neurodermatitides OR Disseminated Neurodermatitis OR Atopic Eczema) AND (dupilumab) AND Random*.

The scientific evidence selected was that which met the following eligibility criteria:

– Population: children and/or adolescents;
– Intervention: dupilumab;
– Comparison: placebo;
– Outcome: efficacy and safety;
– Study design: phase-III randomized clinical trial (RCT); no time or language limits; with full text or abstract with available data.

The data extracted from the selected articles were age, severity of the disease, number of patients who used dupilumab and placebo, dosage and time of application of Dupilumab, follow-up, IGA response, EASI 50 and 75, reduction of pruritus (NRS), and drug-related adverse effects (Table 1).

**Table 1:** Characteristics of the articles included.

First Author	Year	Age	Disease severity	Intervention (Dupilumab)	Comparison	Results	Follow-up
Paller^12^	2022	6 months–6 years	Moderate to severe atopic dermatitis*	Dupilumab 200 to 300 mg SC, 1x every 4 weeks+TCS (n: 83)	Placebo+TCS (same regimen) (n: 79)	IGA response (score of 0 or 1 on the IGA). EASI-75 and response (≥75% improvement in EASI). EASI-50 (≥50% improvement in EASI). NRS (Reduction in itching ≥3 points). TEAEs	16 weeks
Paller (a)^13^	2020	6 years–11 years	Moderate to severe atopic dermatitis ≥1 year*	Dupilumab 100 or 200 mg SC, once every 2 weeks+TCS (n: 122)	Placebo+TCS (same regimen) (n: 123)	IGA response (score of 0 or 1 on the IGA). EASI-75 and response (≥75% improvement in EASI). EASI-50 (≥50% improvement in EASI). NRS (Reduction in itching ≥3 points). TEAEs	28 weeks
Paller b)^13^				Dupilumab 300mg SC, once every 4 weeks+TCS (n: 122)			
Simpson (a)^14^	2020	12–18 years	Moderate to severe atopic dermatitis ≥1 year*	Dupilumab 200mg SC, 1x every 2 weeks (n: 82)	Placebo (same regimen) (n: 85)	IGA response (score of 0 or 1 on the IGA). EASI-75 and response (≥75% improvement in EASI). EASI-50 (≥50% improvement in EASI). NRS (Reduction in itching ≥3 points). TEAEs	28 weeks
Simpson (b)^14^				Dupilumab 300mg SC, 1x every 4 weeks (n: 84)			

* inadequate response to topical corticosteroids.

TCS: topical corticosteroid; SC: subcutaneously; IGA: investigator global assessment; EASI: Eczema Area and Severity Index; NRS: Peak Pruritus Numerical Rating Scale; TEAEs: treatment-emergent adverse events.

The selected evidence was critically assessed by investigating the risk of bias, using RoB 2.0^7^, namely, randomization, blinded allocation, double blinding and evaluator blinding, losses, prognostic characteristics, measurement of outcomes, intention-to-treat analysis, sample calculation, and early interruption. The risk was estimated as very high, high, or low.

The quality of the review of the evidence was expressed as very low, low, moderate, and high. Items considered (using the GRADEpro software)^8^ were rated as very high, high, and low, considering the following criteria: risk of bias, inconsistency, precision, indirect evidence, and publication bias.

The extracted data were meta-analyzed using the RevMan 5.4 software^9^. The results (efficacy and safety) were expressed as risk difference, with a 95% confidence level and figures needed to treat (NNT) or to produce harm (NNH). Heterogeneity in I^2^ ranges from 0 to 100%, with values above 50% considered high (inconsistency). The following models were used: Random (I^2^>50%) and fixed (I^2^≤50%) for analysis.

## RESULTS

**Figure 1:**
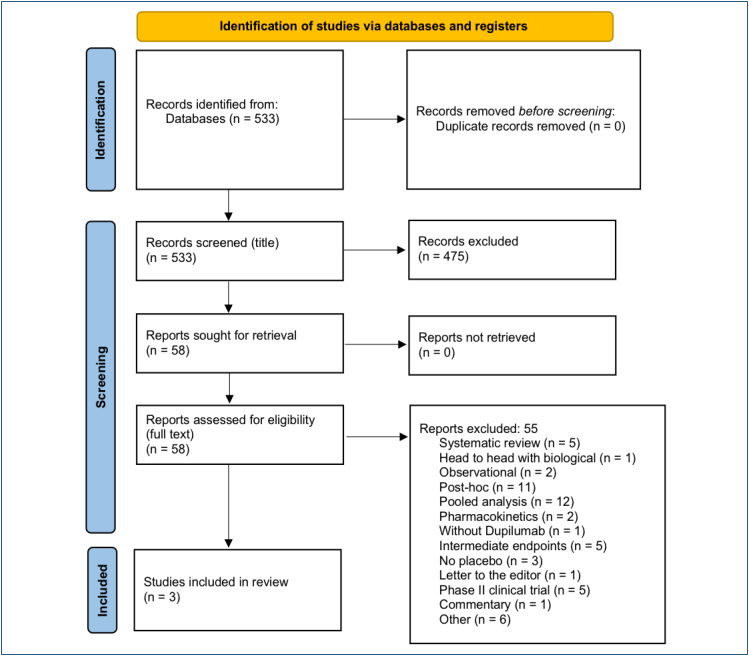
PRISMA flow diagram^5^.

The search, conducted through May 2024, retrieved 533 publications (with limits for RCTs and children), of which 58 were initially selected. Three studies meeting the eligibility criteria were included^1,10,11^ to support this assessment (Figure 1).

The three selected randomized clinical trials included three different age groups: 6 months to 6 years (N: 162)^12^, 6–11 years (N: 367)^13^, and 12–18 years (N: 251)^14^. The total number of patients studied was 780 children and adolescents, 287 of whom received placebo and 493 received dupilumab.

Dupilumab doses ranged from 100 to 300 mg applied subcutaneously (SC) every 2 or 4 weeks for 16 weeks, and follow-up time ranged from 16 to 28 weeks.

The outcomes analyzed were IGA (0–1 and/or improvement ≥2 points), EASI 75 (improvement ≥75%), EASI 50 (improvement ≥50%), NRS pruritus (reduction ≥3 points), and treatment-emergent adverse events (TEAEs).

The risk of bias for one study is low, and for the other two, there are some concerns (Table 2). The quality of the evidence is high (Table 3) for all outcomes analyzed.

**Table 2:** Risk of bias analysis.

		Risk of bias domains					
		D1	D2	D3	D4	D5	Overall
Study	Paller^12^	+	+	+	+	+	+
	Paller^13^	+	+	-	-	+	-
	Simpson^14^	+	+	+	-	+	-
	Domains:					Judgement	
	D1: Bias arising from the randomization process.					-	Some concerns
	D2: Bias due to deviations from intended intervention.					+	Low
	D3: Bias due to missing outcome data.						
	D4: Bias in measurement of the outcome.						
	D5: Bias in selection of the reported result.						

**Table 3:** GRADEPro (certainty of evidence).

Certainty assessment							Nº of patients		Effect		Certainty	Importance
Nº of studies	Study design	Risk of bias	Inconsis­tency	Indirect­ness	Impre­cision	Other considerations	Dupu­limab	Placebo	Relative (95% CI)	Absolute (95% CI)		
IGA (0 a 1 e/ou melhora ≥2 pontos)												
5	Randomised trials	Not serious	Not serious	Not serious	Not serious	None	134/493 (27.2%)	35/495 (7.1%)	RR 3.84 (2.71 to 5.45)	201 more per 1.000 (from 121 more to 315 more)	⊕⊕⊕⊕ High	
EASI 75 (melhora ≥75%)												
5	Randomised trials	Not serious	Not serious	Not serious	Not serious	None	270/493 (54.8%)	76/495 (15.4%)	RR 3.57 (2.86 to 4.45)	395 more per 1.000 (from 286 more to 530 more)	⊕⊕⊕⊕ High	
EASI 50 (melhora ≥50%)												
5	Randomised trials	Not serious	Not serious	Not serious	Not serious	None	365/493 (74.0%)	144/495 (29.1%)	RR 2.54 (2.21 to 2.93)	448 more per 1.000 (from 352 more to 561 more)	⊕⊕⊕⊕ High	
NRS prurido (redução ≥3 pontos)												
5	Randomised trials	Not serious	Not serious	Not serious	Not serious	None	270/493 (54.8%)	76/495 (15.4%)	RR 3.57 (2.86 to 4.45)	395 more per 1.000 (from 286 more to 530 more)	⊕⊕⊕⊕ High	
TEAEs												
5	Randomised trials	Not serious	Not serious	Not serious	Not serious	None	3/493 (0.6%)	7/495 (1.4%)	RR 0.53 (0.17 to 1.66)	7 fewer per 1.000 (from 12 fewer to 9 more)	⊕⊕⊕⊕ High	

CI: confidence interval; RR: risk ratio; IGA: investigator global assessment; EASI: Eczema Area and Severity Index; NRS: Peak Pruritus Numerical Rating Scale; TEAEs: treatment-emergent adverse events..

The results of the three studies were meta-analyzed by aggregating the results of the three age groups since these results were homogeneous in the comparison between dupilumab and placebo, in all the outcomes reviewed.

## RESULTS BY OUTCOME

In children (aged 6 months to 11 years) and adolescents (aged 11–18 years) with moderate to severe AD unresponsive to conventional treatment (TCSs), the use of dupilumab compared to placebo produces the following effects:

### Investigator global assessment (0–1 and/or improvement ≥2 points)

**Figure 2:**
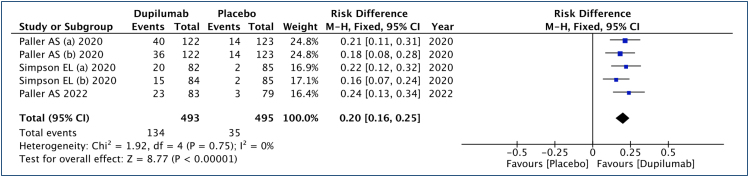
Analysis of the investigator global assessment results in children and adolescents with moderate to severe atopic dermatitis treated with dupilumab.

In this analysis (Figure 2), 493 patients submitted to dupilumab and 495 to the placebo group were studied. An improvement of 20% in IGA scores was observed (range: 16–25%, 95% CI), requiring 5 children or adolescents to be treated to achieve an improvement of ≥2 points or a score between 0 and 1 (NNT: 5). The quality of the evidence is high.

### Eczema Area and Severity Index 75 (improvement ≥75%)

**Figure 3:**
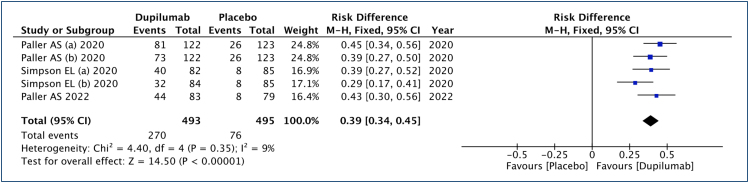
Analysis of the results of Eczema Area and Severity Index 75 in children and adolescents with moderate to severe atopic dermatitis treated with dupilumab.

In this analysis (Figure 3), 493 patients submitted to dupilumab and 495 to the placebo group were studied. An improvement of 39% in EASI 75 scores was observed (range: 33–44%, 95% CI), with an NNT of 3 to achieve a ≥75% improvement in one child or adolescent. The quality of the evidence is high.

### Eczema Area and Severity Index 50 (improvement ≥50%)

**Figure 4:**
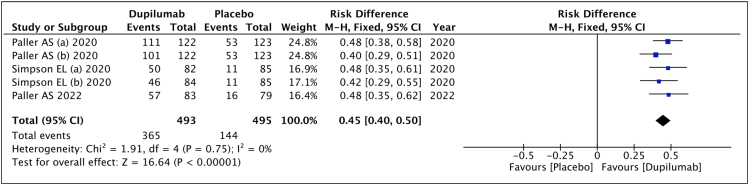
Analysis of the results of Eczema Area and Severity Index 50 in children and adolescents with moderate to severe atopic dermatitis treated with dupilumab.

In this analysis (Figure 4), 493 patients submitted to dupilumab and 495 to the placebo group were studied. An improvement of 45% in EASI 50 scores was observed (range: 40–50%, 95% CI), with an NNT of approximately 2 to achieve a ≥50% improvement in one child or adolescent. The quality of the evidence is high.

### Numerical Rating Scale pruritus (reduction ≥3 points)

**Figure 5:**
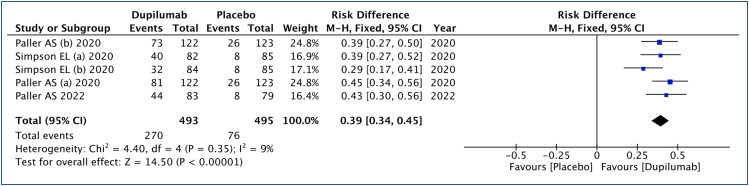
Analysis of the Numerical Rating Scale results in children and adolescents with moderate to severe atopic dermatitis treated with dupilumab.

In this analysis (Figure 5), 493 patients submitted to dupilumab and 495 to the placebo group were studied. A 39% reduction in pruritus (measured by NRS) was observed (range: 34–45%), with an NNT of 3 to achieve a ≥3-point reduction in one child or adolescent. The quality of the evidence is high.

#### Treatment-emergent adverse events

**Figure 6:**
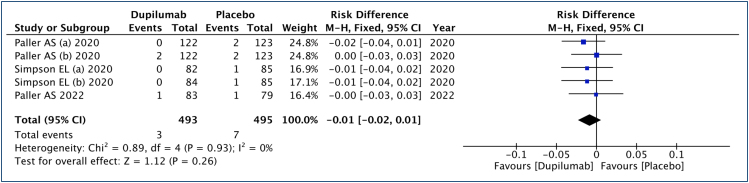
Analysis of the treatment-emergent adverse events (TEA) results in children and adolescents with moderate to severe atopic dermatitis treated with dupilumab.

In this analysis (Figure 6), 493 patients submitted to dupilumab and 495 to the placebo group were studied. There is no difference in the risk of TEAEs with dupilumab treatment compared with placebo. The quality of the evidence is high.

## DISCUSSION

This systematic review and meta-analysis revealed a significant difference between dupilumab and placebo in patients aged 6 months to 18 years with moderate to severe AD. The analysis of outcomes, including scales related to clinical improvement, enhanced quality of life, and lesion improvement, demonstrated the effectiveness of dupilumab. Additionally, this review found no significant difference in adverse effects between dupilumab and placebo, reinforcing the safety of dupilumab for use in children and adolescents. All results presented high-quality evidence and a low risk of bias, with NNTs ranging from 2 to 5 for the different outcomes.

The results highlight that dupilumab is a safe and effective treatment option for children and adolescents with moderate to severe AD who do not respond to conventional treatments. This is a breakthrough for dermatology that offers a safe and effective new approach to managing a chronic debilitating condition. Overall, the approval and use of dupilumab can significantly improve the quality of life of young patients by reducing disease severity and associated impacts, such as ongoing discomfort and comorbidities related to AD without adequate response to topical treatment.

When comparing the results of this study with other similar works, such as the study by Zheng et al.^15^ and the study of Kouwenhoven et al.^16^, it is observed that both corroborate the efficacy of dupilumab in the management of severe AD. Zheng et al.^15^ highlighted the improvement in EASI scores and in the patients’ quality of life, while Kouwenhoven et al.^16^ emphasized the significant reduction in pruritus and the severity of lesions. These results are consistent with those found in the present meta-analysis, reinforcing the clinical relevance of dupilumab in the treatment of AD in pediatric populations.

When comparing the results of this study with data on the use of dupilumab in adults with severe AD, as reviewed by Bernardo et al.^17^, it is observed that the beneficial effects of dupilumab are consistent among different age groups. The values observed in the adult population are comparable to those found in pediatric populations, indicating that dupilumab is equally effective and safe for treating AD across different age groups. The main difference observed was the increased risk of severe AEs in adults, which was not significantly elevated in children, indicating that tolerability may vary with age.

The limitations of this systematic review and meta-analysis are primarily due to the limited amount of available literature, which may impact the result generalizability. Furthermore, clinical trial duration ranged from 16 to 28 weeks, which may not be sufficient to fully assess the long-term effects of dupilumab. Variability in the doses administered (100–300 mg SC every 2 or 4 weeks) may also introduce inconsistencies in the results. Furthermore, it is worth noting that the two studies by Paller et al.^9,13^ used TCS associated with both dupilumab and placebo. Meanwhile, the study by Simpson et al.^14^ at the investigator's discretion tolerated the use of systemic nonsteroidal immunosuppressants, systemic or TCSs, topical calcineurin inhibitors, and topical sodium crisaborole for rescue treatment by patients with intolerable AD symptoms, this factor may have led to interference in the results found by this study.

To strengthen the conclusions, future studies should include a larger number of participants and extend the follow-up period to assess the long-term effects of dupilumab. Furthermore, standardizing the doses administered to reduce variability in results and including studies with different severities of AD would provide a more comprehensive view of the efficacy and safety of dupilumab. Finally, it is worth highlighting the need to conduct more randomized clinical trials that evaluate the use of dupilumab in the pediatric population as a therapeutic option for moderate/severe AD.

## CONCLUSION

In children (age range of 6 months to 11 years) and adolescents (11–18 years of age) with moderate to severe AD unresponsive to conventional treatment, the use of dupilumab is effective and safe.
